# Performance of Aluminum Foam-Filled Hierarchical Thin-Walled Structures Under Axial Impact

**DOI:** 10.3390/ma19102106

**Published:** 2026-05-17

**Authors:** Xinxun Guo, Yaochu Fang, Guoyun Lu, Huiwei Yang, Pengcheng Chen, Jie Zhang

**Affiliations:** 1 College of Civil Engineering, Taiyuan University of Technology, Taiyuan 030024, China; 2 School of Civil Engineering, University of South China, Hengyang 421001, China

**Keywords:** thin-walled structure, aluminum foam, energy absorption, hierarchical design, multi-cell design, theoretical prediction

## Abstract

In this study, a hierarchical aluminum foam-filled thin-walled structure is proposed and its performance under axial impact is subsequently investigated. Two primary configurations are studied, namely, a hierarchical unit-cell structure (HUCS) and hierarchical multi-cell structure (HMS), respectively. Meanwhile, based on the experimental results, models are established to further investigate the effect of geometries, foam densities and impact velocities on the impact performance of the proposed structure. Finally, an improved simplified super folding element (*SSFE*) theoretical model which accounts for the constraint-induced strengthening effect of the foam filler is derived and a closed-form expression for the mean crushing force (*MCF*) is obtained. Compared with non-hierarchical counterparts (NHUCS and NHMS), the hierarchical designs exhibited superiority in reducing deformations and enhancing specific energy absorption (*SEA*). Compared to non-hierarchical structures, under an impact with identical energy, the HUCS shows a 12.7% reduction in maximum deformation and a 15.4% increase in *SEA* at the same deformation level. Meanwhile, the HMS reduces *MCF* by 17.2% and initial peak force (*IPF*) by 20.2% compared to the NHMS. Parametric studies reveal that wall thickness has a greater influence on final deformation than foam density. Numerical results are in good agreement with the proposed *SSFE* model within the baseline parameter range, with typical deviations below 10%, though larger discrepancies up to 23% are observed for certain extreme combinations of wall thickness and foam density. The hierarchical multi-cell collaborative design and the *MCF* prediction method presented here can provide practical guidance for designing high-efficiency impact-protective structures.

## 1. Introduction

With rapid advances in industrial technology, demands for improved impact resistance in sectors such as civil infrastructure, automotive, and aerospace have increased markedly [1]. Under extreme service conditions, impact loads can not only inflict damage on the primary structure but also trigger secondary disasters, leading to significant economic losses and casualties [2,3]. This pressing reality has spurred innovation in the research and development of impact-resistant protective systems, with thin-walled structures—owing to their efficient energy absorption characteristics and economic feasibility—garnering considerable interest in both academic and engineering circles [4,5].

Metallic thin-walled structures have emerged as the core components of impact protection systems due to their excellent specific strength and controllable buckling deformation modes [4]. To overcome the performance limitations of conventional metallic thin-walled structures, researchers have proposed the composite reinforcement of aluminum foam as a functional filler [6,7]. This porous material, with its unique compression hardening effect, significantly enhances the energy dissipation capability of thin-walled structures [8,9,10]. Studies have demonstrated that through a well-conceived composite design, aluminum foam-filled thin-walled structures can achieve energy absorption efficiencies several times higher than those of traditional metallic thin-walled structures [11,12]. Based on this foundation, Li [13] confirmed via quasi-static compression experiments that aluminum foam-filled circular tubes exhibit superior energy absorption performance compared to square tubes; Yin [14] found that, under lateral impact conditions, aluminum foam-filled multi-cell thin-walled structures outperformed their single-cell counterparts in energy absorption; and Lu [15] demonstrated through combined experimental and numerical studies that optimizing the density of aluminum foam could enhance the impact resistance of aluminum foam-filled tubular core sandwich panels. These breakthroughs lay the groundwork for the engineering applications of aluminum foam-filled thin-walled structures.

To further explore the energy absorption potential of aluminum foam-filled thin-walled structures, biomimetic design concepts have been introduced into their development [16,17]. Related research indicates that rational biomimetic design can significantly improve energy absorption performance without increasing the self-weight of the thin-walled structure [18,19]. Building on this, Yao [20] and DucHieu [21] carried out multi-cell designs for bioinspired aluminum foam-filled thin-walled structures, examined their energy absorption performance, and proposed an average crushing force predictive model. Structural optimization remains a key research direction to enhance the energy absorption performance of aluminum foam-filled thin-walled structures [22]. To maximize the advantages of both the aluminum foam material and the thin-walled structure, Fang [23] optimized the energy absorption performance of aluminum foam-filled thin-walled structures based on a Kriging model; meanwhile, Li [24] employed genetic algorithms for multi-objective optimization of their energy absorption performance. Subsequently, Fang [25] further improved energy absorption performance by optimizing the wall thickness gradient and aluminum foam density gradient using a multi-objective particle swarm optimization algorithm based on an established surrogate model.

In the field of innovative structural design, hierarchical design theory has provided a new approach for enhancing energy absorption performance. Hierarchical structures, through multi-scale ordered arrangements, exhibit significant advantages in terms of specific strength, specific stiffness, and controllable failure modes [26,27]. In early research, Kooistra [28] introduced the concept of hierarchy into corrugated sandwich structures by designing a two-level hierarchical folded structure and establishing correspondence between six failure modes and nominal stress based on elastic theory. Subsequently, researchers refined and validated the theoretical models using classical plate theory and Mindlin theory [29,30,31], and further investigated the three-point bending performance of these structures [32]. Additionally, Sun designed a novel hierarchical honeycomb sandwich panel based on hierarchical theory, with studies showing that the dynamic response of this structure under impact and explosive loads significantly outperformed that of conventional honeycomb sandwich panels [33]. Zhang integrated hierarchical design concepts into bioinspired thin-walled structures, proposing a hierarchical fractal thin-walled structure and investigating the effects of fractal order, geometric parameters, and the number of hierarchies on energy absorption performance [34]. These research outcomes have confirmed that hierarchical configurations can effectively regulate buckling behavior and energy dissipation pathways, thereby providing vital insights for the design of novel protective structures [35,36,37,38].

Despite these significant advances in hierarchical structures and foam-filled tubes individually, a critical research gap persists: the synergistic coupling between multi-scale geometric hierarchy and porous foam filling under axial impact has not been systematically characterized, nor has a predictive theoretical framework accounting for this coupling been established. To address this gap, this paper introduces a hierarchical aluminum foam-filled thin-walled structure. The novelty and contributions of this work are explicitly threefold: Novel hierarchical geometry coupled with foam filling: unlike prior work which applies hierarchy to empty shells or foam or simple cross-sections, this study specifically investigates the constraint-induced strengthening effect arising from the interaction between a fractal-like internal wall arrangement (HUCS and HMS) and the lateral expansion of aluminum foam. Rigorous equal-mass comparative framework: to decouple the geometric advantage from the trivial effect of added mass, all performance metrics (*SEA*, *MCF* and Deformation) are evaluated under a strict equal-mass baseline against non-hierarchical counterparts (NHUCS and NHMS). This isolates the intrinsic benefit of hierarchical topology redistribution. Analytical modeling of coupled hierarchical-foam response: an improved simplified super folding element (*SSFE*) theoretical model is derived. The key theoretical advancement is the incorporation of a foam-induced constraint coefficient that modifies the plastic hinge moment specifically within the smaller-scale hierarchical elements, yielding a validated closed-form prediction of mean crushing force (*MCF*) where previous models fail. The main contributions of this work are: (1) the proposal and systematic numerical evaluation of aluminum foam-filled hierarchical unit-cell and multi-cell thin-walled structures under axial impact, (2) derivation of an improved SSFE model that accounts for foam-induced constraint and yields closed-form predictions for the *MCF* of HUCSs and HMSs, (3) parametric studies clarifying the influence of wall thickness, foam density and impact velocity on *SEA*, *MCF* and *IPF*.

## 2. Structural Design

**Figure 1:**
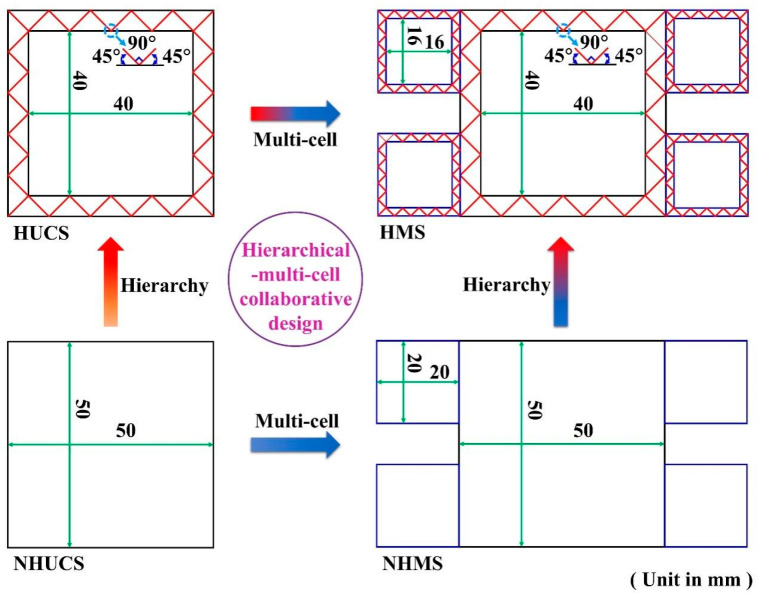
Geometric configurations of NHUCS, HUCS, NHMS and HMS.

To investigate the performance of aluminum foam-filled hierarchical thin-walled structures under low-speed impact conditions, this study designs aluminum foam-filled hierarchical unit-cell and multi-cell structures based on the principles of equal mass and equal area. Their geometrical configurations are shown in Figure 1. Specifically, the aluminum foam-filled hierarchical unit-cell structure comprises an aluminum foam-filled core layer and a hierarchical primary energy-absorbing thin-walled tube, whereas the aluminum foam-filled hierarchical multi-cell structure consists of an aluminum foam-filled core layer, a hierarchical primary energy-absorbing thin-walled tube, and a hierarchical secondary energy-absorbing thin-walled tube. In order to analyze and demonstrate the advantages of the hierarchical and multi-cell designs in enhancing the energy absorption characteristics of the thin-walled structures, for comparison purposes, non-hierarchical thin-walled unit-cell and multi-cell structures filled with aluminum foam are also designed. Their geometrical configurations are depicted in Figure 1. Here, the aluminum foam-filled non-hierarchical unit-cell structure comprises an aluminum foam-filled core layer and a non-hierarchical primary energy-absorbing thin-walled tube, while the aluminum foam-filled non-hierarchical multi-cell structure consists of an aluminum foam-filled core layer, a non-hierarchical primary energy-absorbing thin-walled tube, and a non-hierarchical secondary energy-absorbing thin-walled tube. The aluminum foam-filled hierarchical unit-cell structure is denoted as HUCS, the aluminum foam-filled hierarchical multi-cell structure as HMS, the aluminum foam-filled non-hierarchical unit-cell structure as NHUCS, and the aluminum foam-filled non-hierarchical multi-cell structure as NHMS.

To comparatively study the performance of the NHUCS, NHMS, HUCS, and HMS under axial impact, seven configurations with different parameters are designed, as detailed in Table 1 and Table 2. Specifically, the side length of the NHUCS is 50 mm; for the NHMS, the side length of the non-hierarchical primary energy-absorbing thin-walled tube is 50 mm, and that of the non-hierarchical secondary energy-absorbing thin-walled tube is 20 mm; for the HUCS, the external side length is 50 mm and the internal side length is 40 mm; for and the HMS, the external and internal side lengths of the hierarchical primary energy-absorbing thin-walled tube are 50 mm and 40 mm, respectively, while those of the hierarchical secondary energy-absorbing thin-walled tube are 20 mm and 16 mm, respectively. The baseline configurations (specimens 1) for the NHUCS, HUCS, NHMS, and HMS are designed with equal total mass. The parametric study then explores three primary variables: (1) impact velocity (10, 15, and 20 m/s; specimens 1–3), (2) aluminum foam density (234, 337, and 526 kg/m^3^; specimens 1, 4, 5), and (3) wall thickness, where thickness is varied while holding mass constant across a new set of comparisons (specimens 1, 6, 7). For all cases, the structure height is fixed at 80 mm.

The equal-mass constraint is deliberately chosen as the basis for comparison in this study. In crashworthiness design, the mass of the energy-absorbing system is a critical cost parameter. Therefore, comparing structures of identical mass is a standard and fair methodology to evaluate the efficiency of a new topology (hierarchy) in utilizing material to absorb energy. It is important to acknowledge that this constraint necessitates a redistribution of wall thickness, where the hierarchical structures (HUCS and HMS) have thinner walls over a more complex cross-section compared to their non-hierarchical counterparts (NHUCS and NHMS). Consequently, the observed improvements in *SEA*, *MCF*, and *CFE* are not solely due to the introduction of geometric hierarchy but are a synergistic result of the hierarchical topology and the associated thickness distribution. The subsequent parametric study on wall thickness further isolates the effect of this parameter, demonstrating its dominant role, but the baseline comparison under equal mass provides the most relevant engineering benchmark for assessing the net benefit of adopting a hierarchical design.

## 3. Finite Element Analysis (FEA)

### 3.1. Finite Element Model

**Figure 2:**
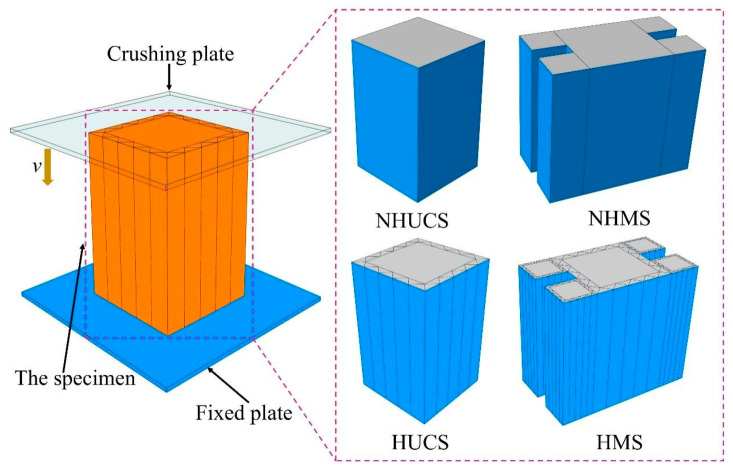
Finite element models.

**Figure 3:**
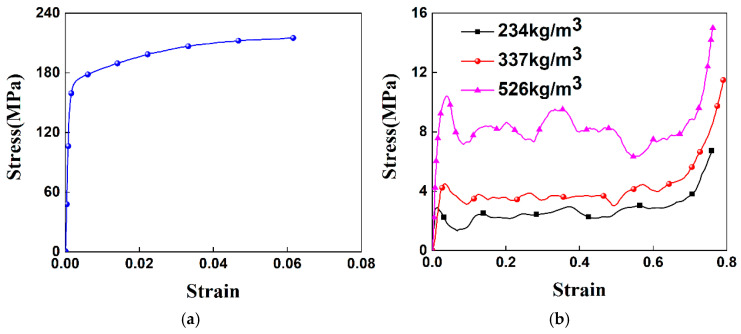
Engineering stress–engineering strain curves [11,12]: (**a**) 6061-T6 aluminum alloy, (**b**) aluminum foam.

As shown in Figure 2, the finite element models of the NHUCS, NHMS, HUCS, and HMS under low-speed axial impact conditions were established using the finite element software ABAQUS 2024. The finite element models of the NHUCS, HUCS, NHMS and HMS consist of four parts: a crush plate, a thin-walled square tube, an aluminum foam-filled core layer, and a fixed plate. Both the non-hierarchical thin-walled tube and the hierarchical thin-walled tube are meshed using S4R shell elements with a mesh size of 1 mm, and five integration points are set in the thickness direction. The aluminum foam-filled core layer is meshed using C3D8R solid elements with a mesh size of 1 mm. The crush plate and fixed plate are modeled as discrete rigid bodies using C3D4 rigid elements with a mesh size of 2 mm. The NHUCS, NHMS, HUCS, and HMS are placed on the fixed plate, and a reference point is set on the crush plate. The reference point mass is set to 100 kg, meaning that the NHUCS, NHMS, HUCS, and HMS are impacted by a 100 kg rigid crush plate moving at velocity *v*. All degrees of freedom of the fixed plate are constrained, with only the crush plate allowed to move in the Z-direction (i.e., axially). The interactions between the thin-walled tube and the rigid plate, the aluminum foam core layer and the thin-walled tube, and the aluminum foam core layer and the rigid plate for the NHUCS, NHMS, HUCS, and HMS during the impact loading process are all modeled using the general contact feature in the ABAQUS interaction module. The coefficient of friction for these interactions is set to 0.3, a value widely adopted in the numerical modeling of aluminum foam-filled tubes [39,40,41]. In the ABAQUS explicit module, the model is calculated and analyzed using explicit dynamic methods. The stress–strain curves of aluminum alloy and aluminum foam are adopted from the constitutive relationship curves for thin-walled aluminum alloy tubes and aluminum foam in quasi-static axial compression tests from the literature [11,12]. The aluminum alloy used is of the 6061-T6 grade, with an elastic modulus of *E* = 68.2 GPa, a Poisson’s ratio of 0.33, and a density of 2710 kg/m^3^. The densities of aluminum foam are 234 kg/m^3^, 337 kg/m^3^, and 526 kg/m^3^, with an elastic modulus of *E* = 80 MPa and a Poisson’s ratio of 0.01. The stress–strain curves for aluminum alloy and foam aluminum at the three densities are shown in Figure 3.

### 3.2. Model Verification

In numerical simulations, to ensure the reliability of the numerical model and the accuracy of the computational results, it is common practice to compare the simulation outcomes with experimental results. Under impact conditions, the reliability of the simulation is typically validated by comparing the deformation patterns of the model, displacement–force curves, and energy variation curves between the simulation and experimental results.

#### 3.2.1. Energy and Mesh Convergence Verification

**Figure 4:**
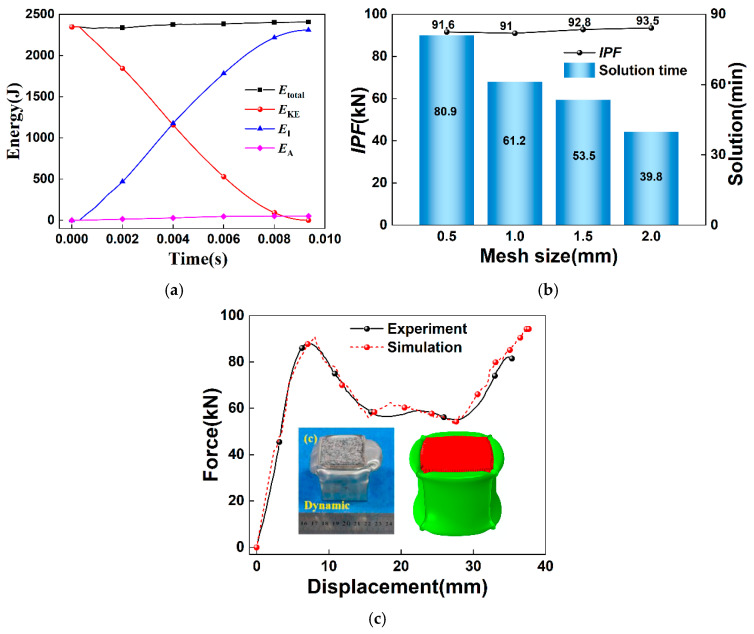
(**a**) Energy–time history curves, (**b**) analysis of mesh convergence, (**c**) comparison of force–displacement curves between the present numerical simulation and the experimental results reported in reference [18] for validation purposes.

In finite element computations, the element size influences both the computational efficiency and the reliability of the results. To ensure the accuracy of the finite element model, it can be observed from the energy–time history curves in Figure 4a that when a mesh with a 1 mm element size is employed, the maximum ratio of the spurious strain energy to the internal energy remains below 5%. Moreover, the total energy (*E*_total_) is approximately constant, with the difference between its maximum and minimum values not exceeding 1%. These findings indicate that the numerical simulation results are highly reliable. In addition to the representative case illustrated in Figure 4a, the hourglass energy was monitored for every simulation presented in this work. Special attention was directed toward the thinnest-walled configurations, specifically the HMS specimens with a wall thickness of 0.4 mm, as these are inherently the most vulnerable to the development of spurious hourglass modes. In all cases examined, the maximum ratio of artificial hourglass energy to internal energy remained consistently below 5% over the entire duration of the impact event. This confirms that the selected 1 mm mesh density, in conjunction with the S4R shell element formulation, provides adequate suppression of zero-energy deformation modes across the full range of geometric and loading parameters investigated. Figure 4b shows the variation in the *IPF* and computation time with respect to four different mesh sizes (0.5 mm, 1 mm, 1.5 mm, and 2 mm). The *IPF* error is smallest when a 1 mm mesh size is used. Based on the analysis presented above, all subsequent numerical simulations adopt a 1 mm mesh size to ensure that the results are both reasonable and dependable. It should be noted that the selection of the 1 mm mesh was based on a combination of the acceptable *IPF* convergence, the low hourglass-to-internal energy ratio (<5%), and the mesh size convention commonly employed in the literature for thin-walled aluminum structures [14,19]. A comprehensive mesh sensitivity study encompassing folding wavelength and energy absorption metrics is encouraged for future high-fidelity investigations.

#### 3.2.2. Verification of Modal and Force–Displacement Curves

To verify the reliability of the numerical simulation results, a simulation of the drop-weight test on an aluminum alloy thin-walled square tube filled with aluminum foam was performed based on the study in reference [18]. The deformation modes from both the experimental and numerical simulation results under the same conditions, as well as the force–displacement curves, are compared, as shown in Figure 4b.

It can be observed from the figure that the deformation mode of the numerical simulation results highly agrees with that of the experiment. In both the numerical simulation and the experiment, the top end (the impact end) of the aluminum alloy thin-walled square tube is the first to experience buckling, followed by progressive axisymmetric folding deformation. Additionally, the folding deformation is quite uniform, and the aluminum foam filling inside the tube undergoes significant compression. The force–displacement curve obtained from the numerical simulation matches well with the experimental results. The initial peak force differs by only 3.4%, and the variation trend of the curve in the platform region is similar, with a platform value deviation of just 5.9%, ensuring the accuracy and validity of the subsequent simulation results. This demonstrates the reliability of the finite element model.

#### 3.2.3. Limitations of the Numerical Model and Implications for the Study

It is crucial to acknowledge the limitations of the present numerical framework. First, the validation of the finite element model relies on a comparative study from the existing literature [18] rather than bespoke experiments conducted on the proposed HUCS and HMS configurations. While this serves as a reasonable verification of the modeling techniques for aluminum foam-filled square tubes, it does not fully validate the predictive accuracy for the more complex hierarchical and multi-cell geometries introduced here. Future work should prioritize experimental testing of the proposed designs for direct model correlation.

Second, the constitutive model for the 6061-T6 aluminum alloy employed here does not incorporate strain rate sensitivity. The validation case and the parametric study cover impact velocities from 10 to 20 m/s. While Langseth [42] suggests that the dynamic enhancement for aluminum alloys in this velocity range is moderate (with a dynamic enhancement factor λ between 1.3 and 1.6), the omission of rate-dependent material properties may lead to an underestimation of the *IPF* and *MCF*, particularly at higher velocities. This limitation is partially addressed in the theoretical model through the incorporation of the empirical dynamic enhancement factor λ, but it remains an inherent simplification in the FEA results. The qualitative trends and comparative performance between structures are expected to remain valid, but absolute quantitative predictions should be interpreted with this caveat.

A more fundamental limitation concerns the validation hierarchy. The current FE modeling methodology was corroborated against axial crushing tests of a conventional square tube [18]. While this provides confidence in the material and contact modeling, a rigorous validation of both the FE and theoretical models for the proposed HUCS and HMS configurations remains absent due to the lack of dedicated experiments. The complex multi-corner folding mechanisms in these hierarchical structures may introduce deformation modes absent in simpler geometries. Consequently, the theoretical predictions presented in Section 6 must be interpreted as having been confirmed against numerical simulations, not physically validated. This represents the most critical gap in the current model maturity, and dedicated experimental testing of the HUCS and HMS is identified as an indispensable next step in future work.

Methodological asymmetry in strain-rate treatment: an important methodological inconsistency exists in the current comparative framework. The FE model, as a material-level simplification, does not incorporate strain-rate sensitivity for the 6061-T6 alloy, thus effectively modeling a quasi-static material response under dynamic loading. Conversely, the theoretical model explicitly accounts for dynamic enhancement through the empirical factor λ. The comparison in Section 6 is therefore between a quasi-static material FEA and a dynamic theory. The reasonable agreement observed suggests that, for the range of velocities and geometries studied, the kinematic constraint effects of hierarchy might dominate the dynamic material effects. However, this agreement cannot be interpreted as a rigorous validation. It highlights a critical need for future high-fidelity simulations and experiments that incorporate rate-dependent constitutive models (e.g., Johnson–Cook) to establish a methodologically consistent and fully validated predictive framework.

## 4. Results

### 4.1. Dynamic Behavior

**Figure 5:**
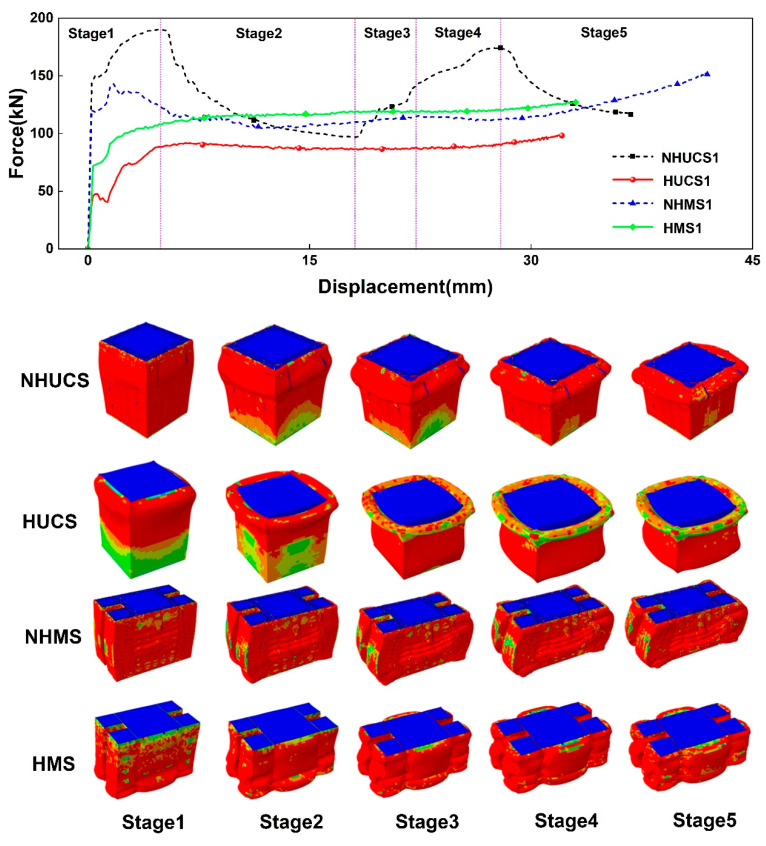
Force–displacement curves and deformation patterns of NHUCS-1, HUCS-1, NHMS-1 and HMS-1 of the same mass at the same impact velocity.

By comparing the force–displacement curves and deformation modes of the four structures with the same mass under the same impact conditions (10 m/s) as shown in Figure 5, it can be observed that all four structures exhibit progressive axisymmetric wrinkling failure modes under impact loading. However, for the NHUCS, the deformation mode is characterized by buckling deformation of the aluminum alloy thin-walled structure, which symmetrically flips outward to form wrinkles. The wavelength of the wrinkles is relatively large, with more folding deformation, and the force–displacement curve fluctuates significantly, indicating poor deformation stability. For the HUCS, the deformation mode is the buckling of the aluminum alloy thin-walled structure, which symmetrically bulges outward and forms a certain degree of wrinkles. The wrinkle wavelength is smaller, the plastic deformation area is larger, and the total displacement is the smallest. In the case of the NHMS, the wrinkle wavelength is relatively large with more folding deformation. The buckling deformation of the aluminum alloy thin-walled structure causes both the primary and secondary energy absorption structures to undergo significant axisymmetric outward bulging deformation. From the force–displacement curve, it is evident that the total deformation displacement is the largest, the force–displacement curve fluctuates more, and its energy absorption efficiency is the lowest, indicating poor crashworthiness under this working condition. For the HMS, the aluminum alloy thin-walled structure undergoes buckling deformation, and both the primary and secondary energy absorption structures form numerous small wrinkles with smaller axisymmetric outward bulging deformations. The total deformation displacement is smaller, and the force–displacement curve exhibits the least fluctuation and the most stable variation.

### 4.2. Crashworthiness Indices

To evaluate the energy absorption performance of the structure, it is essential to introduce some crashworthiness indices to evaluate the energy absorption capacity of the structure. This paper primarily introduces the following indices to assess the energy absorption performance of the structure:

(1) Total energy absorption, *EA*

Total energy absorption refers to the total amount of energy absorbed by the structure during the impact process. For energy-absorbing structures, a higher total energy absorption indicates stronger energy absorption capability. The total energy absorption is calculated using the following formula:

(1) $$EA=\int_{0}^{\eta }F(x)dx$$

where *F*(*x*) represents the impact load at a certain moment, η denotes the impact displacement, and *EA* is the total energy absorption.

(2) Specific energy absorption, *SEA*

Specific energy absorption is the energy absorbed per unit mass of the structure, reflecting the material efficiency in the energy absorption process during the impact. For energy-absorbing structures, a higher specific energy absorption implies stronger energy absorption capability. The specific energy absorption is calculated as follows:

(2) $$SEA=\frac{EA}{M}$$

where *EA* is the total energy absorption of the structure, and *M* is the total mass of the structure.

(3) Initial peak force (*IPF*), *F*_max_

The initial peak force refers to the first impact force peak at the beginning of the impact stage. An excessively high initial peak force can cause a large acceleration. Therefore, for energy-absorbing structures, a lower initial peak force is preferable.

(4) Mean crushing force (*MCF*), *F*_mean_

The mean crushing force is an indicator that represents the energy absorption capacity of the structure per unit crushing distance. It is calculated as follows:

(3) $$F_{mean}=\frac{EA}{\eta }$$

where *EA* is the total energy absorption of the structure, and η is the crushing distance.

(5) Crushing force efficiency, *CFE*

Crushing force efficiency is an effective evaluation parameter for load consistency during the impact process. It is defined as the ratio of the mean force to the initial peak force, calculated using the formula

(4) $$CFE=\frac{F_{mean}}{F_{\max}}\times 100\%$$

A higher crushing force efficiency indicates stronger energy absorption capacity of the structure.

These crashworthiness parameters provide a comprehensive evaluation of the energy absorption performance of structures under impact loading, and they are critical for understanding and improving the design of energy-absorbing materials and structures.

**Figure 6:**
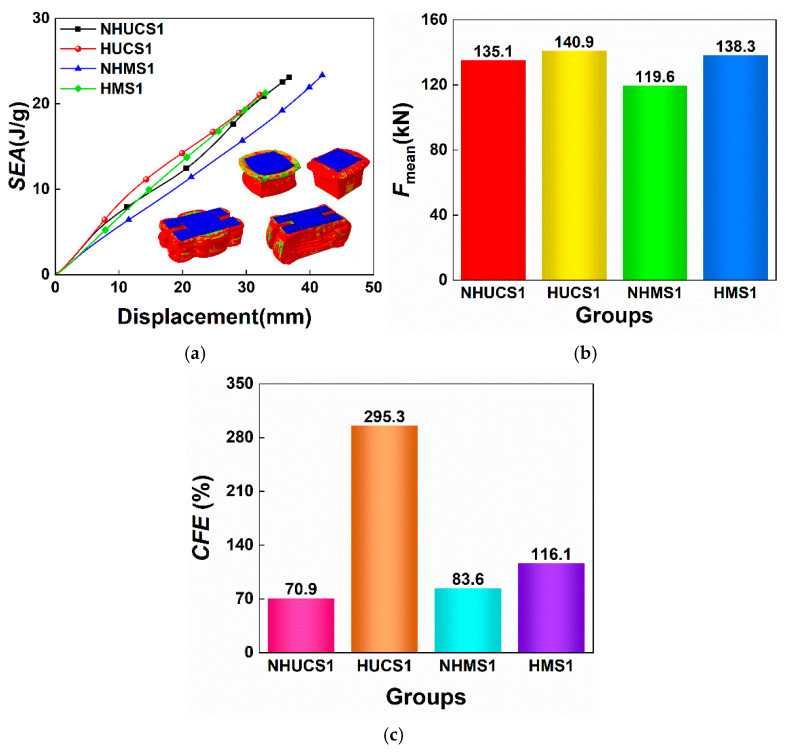
NHUCS-1, HUCS-1, NHMS-1 and HMS-1 of the same mass at the same impact velocity: (**a**) *SEA*–displacement curves, (**b**) *F*_mean_, (**c**) *CFE*.

**Figure 7:**
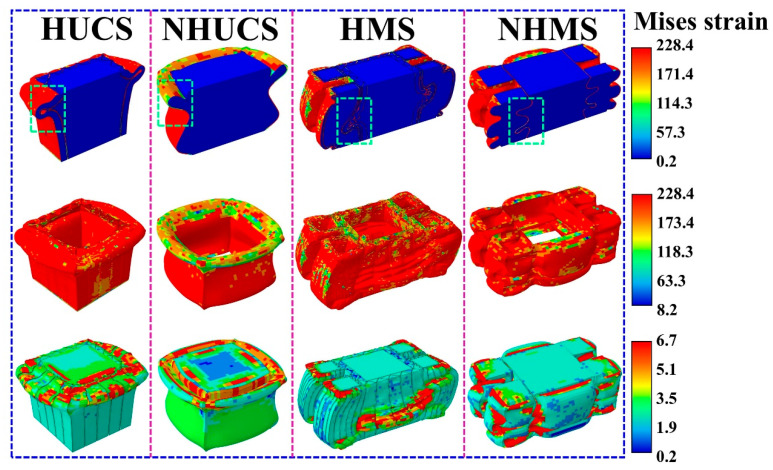
Stress profiles of NHUCS-1, HUCS-1, NHMS-1 and HMS-1 of the same mass for the same impact condition.

The *SEA*–displacement curves and *MCF* and *CFE* bar charts of these four structures under the same impact conditions are shown in Figure 6. From the *SEA*–displacement curves, it can be observed that, for the same deformation displacement, the *SEA* values of the HUCS and HMS are both greater than those of the NHUCS and NHMS. This is attributed to the hierarchical design, which increases the stiffness of the aluminum alloy thin-walled structure, enhancing its deformation resistance and impact performance. As a result, the total deformation of the hierarchical structures is smaller than that of the non-hierarchical structures under the same impact energy. Figure 7 shows the stress contour maps of these four structures under the same impact conditions. By comparing the stress contour maps of the HUCS and HMS, it can be observed that under the same impact load, deformation in the HUCS occurs at the upper part of the structure, where the degree of folding deformation is more significant. In contrast, deformation in the HMS occurs at the lower part of the structure, with a relatively smaller degree of folding deformation. This is due to the multi-cell design of the HMS, which increases the bearing surface area and alters the load transfer path. During the impact load application, energy absorption is facilitated through the interaction of the primary and secondary energy-absorbing structures, resulting in less folding deformation in the HMS. From the above analysis, it can be concluded that, under the same impact conditions, aluminum foam-filled thin-walled structures with hierarchical and multi-cell designs effectively enhance their energy absorption performance while maintaining the same mass.

A particularly noteworthy observation from Table 3 is the exceptionally high *CFE* of HUCS1, reaching 295.3%, which implies that the mean crushing force is nearly three times the initial peak force. This counterintuitive result can be understood by examining the force–displacement curve and deformation mode of HUCS1 in Figure 5. The hierarchical design introduces multiple small-scale corner elements and internal webs that significantly reduce the initial buckling resistance compared to a monolithic thick-walled tube. An *IPF* of only 47.7 kN corresponds to the local yielding and initial folding of these thinner sub-elements. Once this initial fold is initiated, the structure rapidly transitions into a progressive folding mode where multiple plastic hinges form simultaneously across the hierarchical network. This distributed yielding mechanism causes the crushing force to rise and stabilize at a much higher plateau level. In essence, the hierarchical configuration decouples the initial collapse trigger from the subsequent steady-state energy absorption, yielding an exceptionally low *IPF* relative to the sustained crushing resistance. This characteristic is highly desirable in crashworthiness applications, as it minimizes peak acceleration while maintaining high energy absorption capacity.

## 5. Discussion

In the study of energy absorption performance of thin-walled structures filled with aluminum foam, the wall thickness, impact velocity, and aluminum foam density are frequently the focus of researchers. Therefore, it is essential to investigate the influence of these three factors on the energy absorption of thin-walled structures with foam aluminum-filled layers. Table 3 and Table 4 show the numerical simulation results of the four structures under different impact conditions.

### 5.1. Wall Thickness

**Figure 8:**
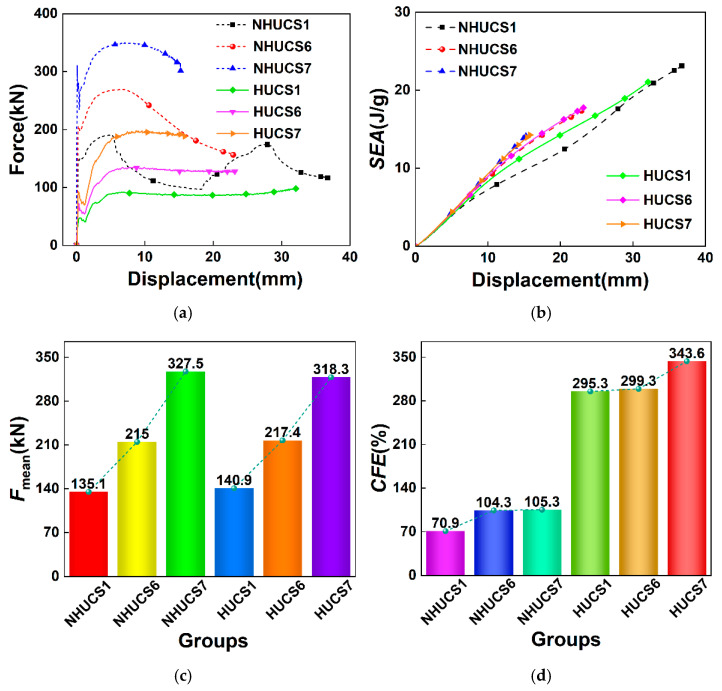
Results of NHUCS and HUCS with different wall thickness parameters at same impact velocity: (**a**) force–displacement curves, (**b**) *SEA*–displacement curves, (**c**) *F*_mean_, (**d**) *CFE*.

It is important to distinguish between two types of comparisons presented in this section. The baseline comparison (specimens 1 in Table 1 and Table 2) is conducted under strict equal-mass conditions, allowing for a fair evaluation of the net benefit of hierarchical design. In contrast, the parametric study on wall thickness (specimens 1, 6, and 7) intentionally varies both thickness and mass to isolate the influence of this geometric parameter on structural response. The force–displacement curves, *SEA*–displacement curves, and *MCF* and *CFE* bar charts of the NHUCS and HUCS under different wall thickness parameters are shown in Figure 8. From the force–displacement curve, it can be observed that, regardless of whether it is a NHUCS or HUCS, the thicker the wall, the more stable the fluctuations in the force–displacement curve, and the smaller the total deformation displacement. This indicates that, with an increase in wall thickness, the energy absorption performance of both the NHUCS and HUCS improves. However, it is also evident that, for the same mass, the platform force of the force–displacement curve of the HUCS is lower than that of the NHUCS. At the same impact energy, the thicker the wall, the greater the *MCF* of the HUCS. It is also found that for the same mass, the *MCF* of the NHUCS and HUCS is very similar. At the same impact deformation displacement, for the same mass, the HUCS exhibits higher *SEA* values and *CFE* compared to the NHUCS. This further confirms that the hierarchical design indeed enhances the energy absorption of the HUCS.

**Figure 9:**
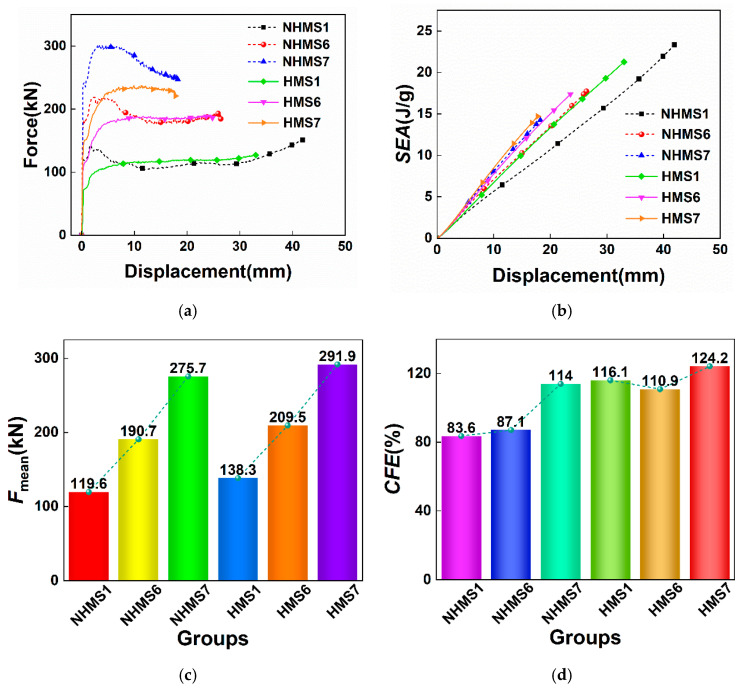
Results of NHMS and HMS with different wall thickness parameters at same impact velocity: (**a**) force–displacement curves, (**b**) *SEA*–displacement curves, (**c**) *F*_mean_, (**d**) *CFE*.

The force–displacement curves, *SEA*–displacement curves, and *MCF* and *CFE* bar charts of the NHMS and HMS under different wall thickness parameters are shown in Figure 9. From the force–displacement curve, it can be seen that the force–displacement curve fluctuations of the NHMS and HMS are more stable than those of the NHUCS and HUCS. This stabilization is a direct consequence of the increased plastic bending moment capacity of the tube walls, which provides greater resistance to the initiation and propagation of non-axisymmetric buckling lobes. This is because the multi-cell design increases the stiffness and load-bearing area of the thin-walled structure. Therefore, under the same impact load, the total deformation displacement of the multi-cell structure is smaller than that of the unit-cell structure with the same mass. Moreover, with an increase in wall thickness, the mass of the multi-cell structure also increases, leading to a reduction in total deformation displacement. For example, in the case of the HMS, when the wall thickness increases from 0.4 mm to 0.8 mm, the total deformation displacement decreases from 33 mm to 17.8 mm. This shows that the wall thickness parameter significantly influences the energy absorption of the hierarchical structure. From the *SEA*–displacement curve, it can be observed that for both the NHMS and HMS, as the wall thickness increases, the *SEA* value of the multi-cell structure increases. The bar charts of *MCF* and *CFE* also show that, with an increase in wall thickness, both the *MCF* and *CFE* improve. This indicates that appropriately increasing the wall thickness can enhance the energy absorption of the multi-cell structure. However, it is also noted that for the same deformation displacement, for the same mass, the HMS exhibits higher *SEA*, *MCF*, and *CFE* compared to the NHMS. This further confirms that the energy absorption of the aluminum foam-filled thin-walled multi-cell structure can be significantly improved after hierarchical design.

### 5.2. Impact Velocity

**Figure 10:**
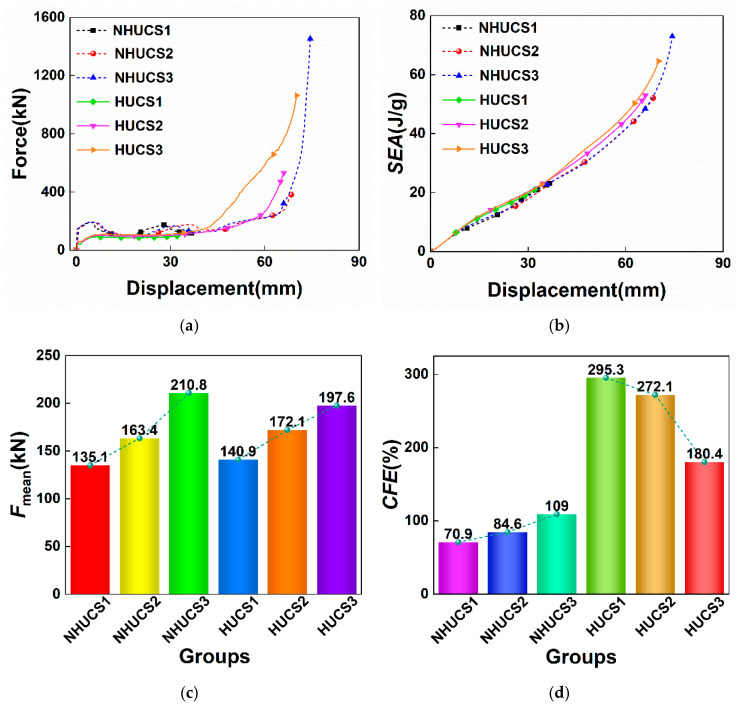
Results for the same mass of the NHUCS and HUCS at different impact velocities: (**a**) force-displacement curves, (**b**) *SEA*–displacement curves, (**c**) *F*_mean_, (**d**) *CFE*.

To investigate the effects of different axial impact velocities on the energy absorption of aluminum foam-filled hierarchical thin-walled structures, three axial impact conditions with velocities of 10 m/s, 15 m/s, and 20 m/s were designed. Figure 10 presents the force–displacement curves, *SEA*–displacement curves, and *MCF* and *CFE* bar charts for the NHUCS and HUCS with equal mass of aluminum foam filling. It can be observed that under all three impact velocities, the force–displacement curves for the HUCS are very smooth with minimal fluctuations, whereas the NHUCS exhibits considerable fluctuations during the plateau phase, indicating that the HUCS has superior deformation stability compared to the NHUCS. From the *SEA*–displacement curves, although the *SEA* for the HUCS and NHUCS is quite similar for identical deformation displacements under each impact velocity, the maximum deformation displacement of the HUCS is consistently lower than that of the NHUCS at each impact velocity. Specifically, at impact velocities of 10 m/s, 15 m/s, and 20 m/s, the maximum deformation displacements for the HUCS are 32 mm, 66 mm, and 70.2 mm, respectively, compared to 36.8 mm, 68.4 mm, and 74.5 mm for the NHUCS. Furthermore, when comparing the *MCF* and *CFE* of the HUCS and NHUCS under the three impact speeds, the HUCS demonstrates higher values for both parameters. As the impact speed increases, the *MCF* and *CFE* of the NHUCS also increase. In contrast, while the *MCF* of the HUCS increases with the impact speed, its *CFE* decreases. This decrease is attributed to the fact that the *IPF* of the HUCS increases with impact speed (47.7 kN, 63.3 kN, and 109.5 kN for 10 m/s, 15 m/s, and 20 m/s, respectively), and although the increase in impact kinetic energy causes the overall structure’s *MCF* to rise, the difference in the *MCF* across the conditions is marginal, thereby reducing the *CFE*. This suggests that for the unit-cell hierarchical design, the inertial stabilization effect at higher velocities is less effective at suppressing the initial peak compared to its influence on the subsequent progressive crushing.

**Figure 11:**
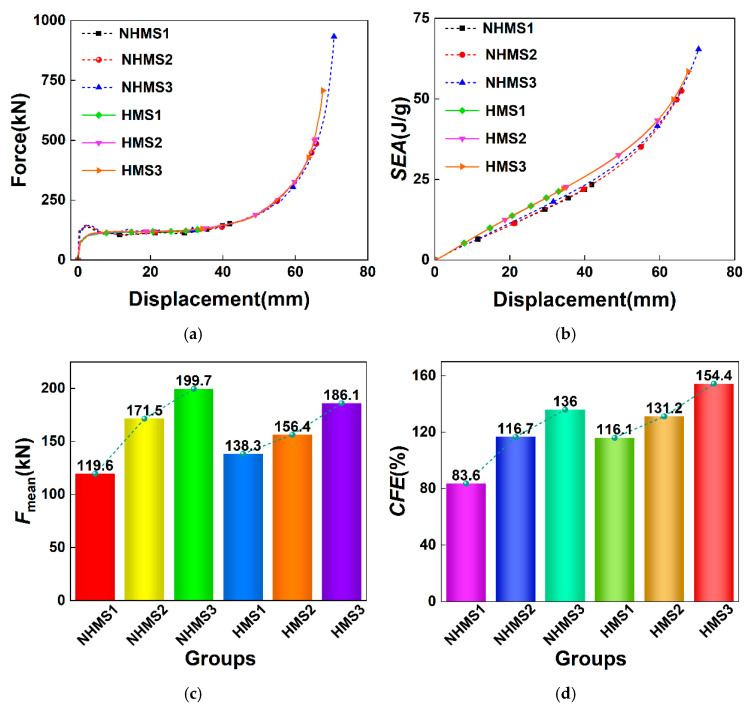
Results for the same mass of the NHMS and HMS at different impact velocities: (**a**) force-displacement curves, (**b**) *SEA*–displacement curves, (**c**) *F*_mean_, (**d**) *CFE*.

For the HMS, Figure 11 illustrates the force–displacement curves, *SEA*–displacement curves, and *MCF* and *CFE* bar charts under the three impact velocities. It is evident that following the multi-cell design, the force–displacement curves of the HMS are very smooth under all impact velocities. However, for each impact condition, both the *IPF* and the maximum deformation displacement of the HMS are notably lower than those of the NHMS. For the same deformation displacement, the HMS exhibits a higher *SEA* compared to the NHMS. Furthermore, with the increase in impact speed, the *MCF* and *CFE* of both the HMS and NHMS increase, but for each impact velocity, the *MCF* and *CFE* of the HMS are higher than those of the NHMS. This indicates that for the same impact conditions, the hierarchical design can enhance the energy absorption of thin-walled multi-cell structures with equal mass.

### 5.3. Density of Aluminum Foam

**Figure 12:**
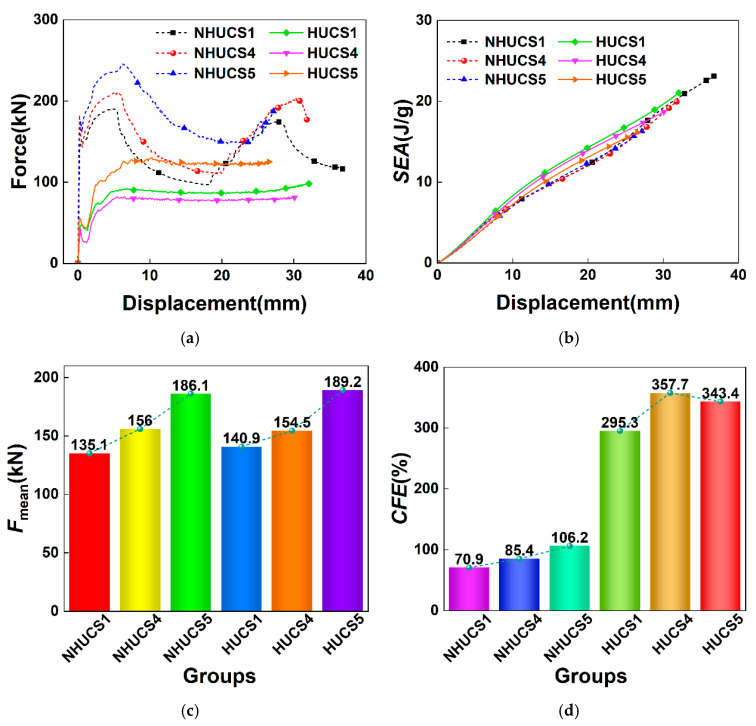
Results of the NHUCS and HUCS filled with different densities of aluminum foam at the same impact velocity: (**a**) force-displacement curves, (**b**) *SEA*–displacement curves, (**c**) *F*_mean_, (**d**) *CFE*.

According to previous studies, the density of aluminum foam is one of the influential factors affecting the energy absorption performance of thin-walled structures. To investigate the effect of aluminum foam density on the energy absorption of thin-walled structures, aluminum foam with densities of 234 kg/m^3^, 337 kg/m^3^, and 526 kg/m^3^ was used to fill thin-walled unit-cell and multi-cell structures with identical wall thicknesses. The crash responses of these structures were analyzed under an impact velocity of 10 m/s. Figure 12 presents the force–displacement curves, *SEA*–displacement curves, and bar charts of *MCF* and *CFE* for the NHUCS and HUCS at an impact velocity of 10 m/s.

From the force–displacement curves, it can be observed that as the density of the aluminum foam increases, both the *IPF* and plateau force of the NHUCS and HUCS increase. However, the force–displacement curves of the HUCS exhibit a significantly smoother trend compared to those of the NHUCS. Regardless of the foam density, the maximum deformation displacement of the HUCS is consistently smaller than that of the NHUCS. When the foam density increases from 234 kg/m^3^ to 337 kg/m^3^, a 44% increase, the *IPF* of the NHUCS decreases from 190.4 kN to 182.8 kN, a reduction of 4%, while that of the HUCS decreases from 47.7 kN to 43.2 kN, a 9.4% reduction. When the foam density increases from 234 kg/m^3^ to 526 kg/m^3^, a 124.8% increase, the *IPF* of the NHUCS decreases to 175.2 kN (a reduction of 8%), whereas that of the HUCS increases to 55.1 kN (an increase of 15.5%). These results suggest that when the foam density is increased by 125%, the *IPF* of the NHUCS is reduced to 92% of its original value, resulting in only an 8% improvement in energy absorption. In contrast, the *IPF* of the HUCS increases to 115% of its original value, indicating a decrease in energy absorption, albeit by only 15.5%. This implies that under the same impact conditions, variations in aluminum foam density have a limited effect on the energy absorption of both the NHUCS and HUCS.

From the *SEA*–displacement curves, it can be seen that under identical impact conditions, the *SEA* values of the NHUCS and HUCS are very close across all three foam densities. The bar charts of *MCF* and *CFE* further indicate that the HUCS consistently outperforms the NHUCS in terms of both metrics across all foam densities, highlighting the advantage of hierarchical design in energy absorption. Specifically, when the foam density is 234 kg/m^3^, the *MCF* and *CFE* of the NHUCS are 135.1 kN and 70.9%, respectively, compared to 140.9 kN and 295.3% for the HUCS. When the foam density increases to 526 kg/m^3^, the *MCF* and *CFE* of the NHUCS rise to 186.1 kN and 106.2%, while those of the HUCS increase to 189.2 kN and 343.4%. This indicates that when the foam density is increased by 125%, the *MCF* and *CFE* of the NHUCS increase by only 37.7% and 35.3%, respectively, while those of the HUCS increase by 34.3% and 48.1%, respectively. These results demonstrate that under the same impact conditions, increasing the foam density has limited impact on improving the energy absorption of both the NHUCS and HUCS, suggesting that enhancing energy absorption by increasing foam density is not cost-effective.

**Figure 13:**
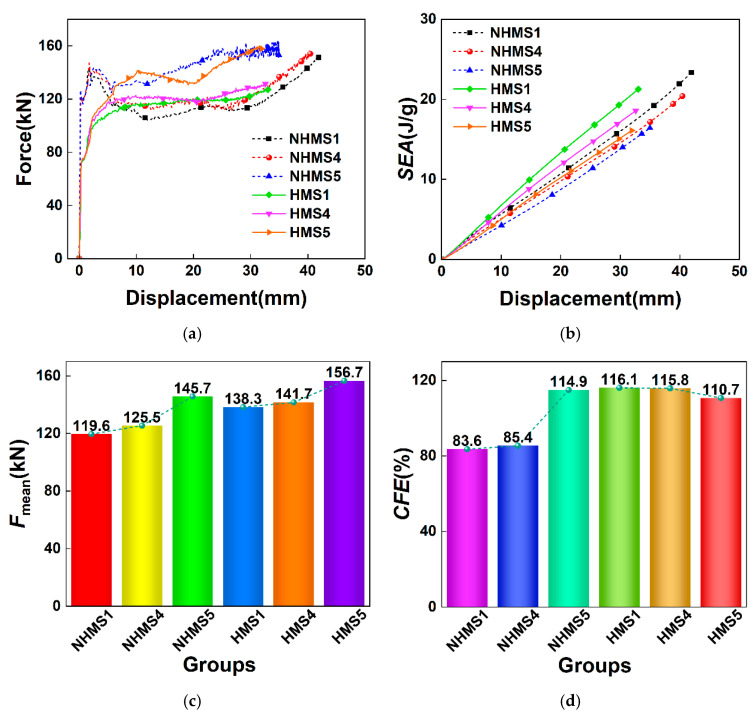
Results of the NHMS and HMS filled with different densities of aluminum foam at the same impact velocity: (**a**) force-displacement curves, (**b**) *SEA*–displacement curves, (**c**) *F*_mean_, (**d**) *CFE*.

Figure 13 shows the force–displacement curves, *SEA*–displacement curves, and bar charts of *MCF* and *CFE* for the NHMS and HMS under an impact velocity of 10 m/s. From the force–displacement curves, it can be observed that the plateau force of both the NHMS and HMS increase with increasing foam density, while their maximum deformation displacements decrease. This indicates that increasing the foam density enhances the energy absorption of both the NHMS and HMS. Moreover, for the same foam density, the maximum deformation displacement of the HMS is consistently smaller than that of the NHMS. The *SEA*–displacement curves and the bar charts further show that, under the same deformation displacement, the HMS consistently achieves higher *SEA* values than the NHMS. For a given foam density, the HMS also exhibits higher *MCF* and *CFE* compared to the NHMS, confirming the superiority of hierarchical design in enhancing the energy absorption of foam-filled thin-walled multi-cell structures.

Specifically, when the foam density is 234 kg/m^3^, the *MCF* and *CFE* of the NHMS are 119.6 kN and 83.6%, respectively, compared to 138.3 kN and 116.1% for the HMS. When the foam density increases to 526 kg/m^3^, the *MCF* and *CFE* of the NHMS rise to 145.7 kN and 114.9%, while those of the HMS increase to 156.7 kN and 110.7%. Therefore, when the foam density is increased by 125%, the *MCF* and *CFE* of the NHMS increase by only 21.8% and 31.3%, respectively, while the *MCF* of the HMS increases by merely 13.3%, and its *CFE* even decreases by 5.4%. Compared to the NHUCS and HUCS, the influence of foam density on the energy absorption of the NHMS and HMS is even smaller, further substantiating that increasing foam density is a relatively low-cost-effective strategy for improving the energy absorption of such structures.

## 6. Theoretical Analysis

As mentioned previously, both the HUCS and HMS exhibit exceptional energy absorption and crashworthiness. The simplified super folding element (*SSFE*) theory has gained widespread recognition for its capability to analyze the energy absorption characteristics of such structures [35,36,37]. Therefore, this section proposes a theoretical model for predicting the *MCF* based on the *SSFE* theory and foam filling theory. It is hypothesized that the folding wavelength (2*H*) of each wall remains constant during the progressive folding deformation of the HUCS and HMS. The system’s energy balance can then be established to determine the *MCF*. Specifically, the external work performed by the crushing force should balance the internal energy dissipated through wall bending, wall stretching, and foam material compression during structural collapse. Consequently, the energy balance equation for the system can be expressed as

(5) $$F_{mean}\cdot 2H\cdot k=E_{b}+E_{m}$$

In the equation, *E*_b_ and *E*_m_ denote the bending energy and membrane energy, respectively; 2*H* represents the folding wavelength; and *k* is the effective crushing distance coefficient. According to Abramowicz and Jones [38], the actual folding wavelength is smaller than 2*H* due to incomplete wall flattening.

### 6.1. Bending Energy

**Figure 14:**
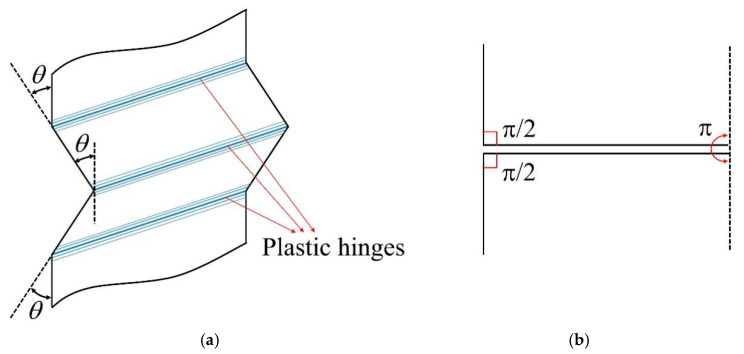
Schematic illustration of flange deformation under the crushing process: (**a**) bending deformation characterized by the rotation angle denoted as *θ*, (**b**) showcase of the rotation angles at three hinges subsequent to the crushing process, which assume values of *π/2*, *π*, and *π/2* [39].

The total bending energy per folding during the crushing process is obtained by summing the energy dissipation across three plastic stationary hinge lines [39], as illustrated in Figure 14a. Therefore, the bending energy can be determined as

(6) $$E_{b}=\sum_{i=1}^{3}M_{o}\theta_{i}L_{c}$$

In the equation, $L_{c}$ represents the total length of all flanges, $\theta_{i}$ denotes the rotation angle around the i-th plastic hinge, and $M_{o}$ is the fully plastic bending moment per unit length of the square tube. The formula for calculating $M_{o}$ is given by

(7) $$M_{o}=\frac{\sigma_{o}t^{2}}{4}$$

In the equation, $\sigma_{o}$ represents the flow stress of the thin-walled square tube material, and t denotes the wall thickness of the tube. The flow stress $\sigma_{o}$ is calculated as

(8) $$\sigma_{o}=\sqrt{\frac{\sigma_{y}\sigma_{u}}{1+\mu }}$$

In the equation, $\sigma_{y}$ denotes the yield stress of the thin-walled square tube material, $\sigma_{u}$ represents the ultimate stress, and μ is the strain hardening exponent. For 6061-T6 aluminum alloy, the strain hardening exponent μ is taken as 0.05.

Assuming complete flattening of the three hinge lines after crushing, the rotation angles of the hinges are π/2, π and π/2, as illustrated in Figure 14b. The bending energy in Equation (2) can then be expressed in the following form:

(9) $$E_{b}=2\pi M_{o}L_{c}$$

### 6.2. Membrane Energy

In this section, the membrane energy of three-panel right-angle elements, K-shaped four-panel corner elements, five-panel corner elements, and six-panel corner elements in hierarchical thin-walled structures is analyzed.

#### 6.2.1. Three-Panel Right-Angle Element

Zhang [40] conducted theoretical and numerical studies on the energy absorption performance of three-panel right-angle elements. The membrane energy of a three-panel right-angle element per folding wavelength is expressed as

(10) $$E_{m1}=\frac{4M_{o}H^{2}}{t}(1+2\tan\frac{\theta_{1}}{2})$$

#### 6.2.2. K-Shaped Four-Panel Corner Element

The K-shaped four-panel corner element in hierarchical thin-walled structures consists of four flat panels. The folding behavior of the structure governs membrane energy dissipation, which primarily occurs at the intersection regions of the corner elements and depends predominantly on the angles between adjacent panels. The membrane energy per folding wavelength for a K-shaped four-panel corner element is given by [41]

(11) $$E_{m2}=\frac{8M_{o}H^{2}}{t}(1+\frac{1}{\cos\theta_{2}})$$

#### 6.2.3. Five-Panel Corner Element

**Figure 15:**
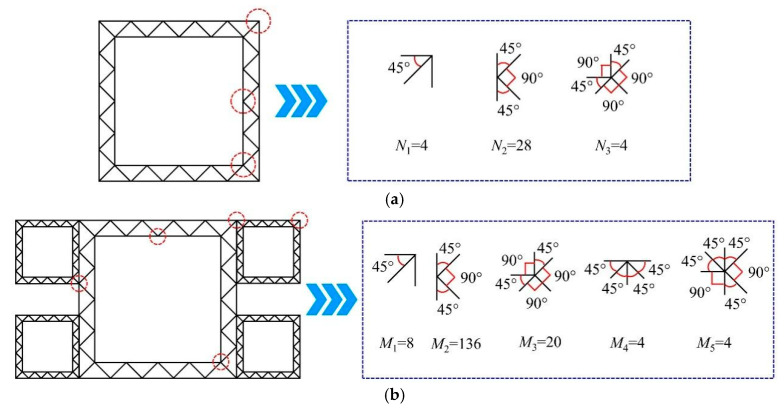
Basic corner elements of the hierarchical thin-walled structures: (**a**) hierarchical thin-walled unit-cell structure (HUCS), (**b**) hierarchical thin-walled multi-cell structure (HMS).

In the HUCS, there exists only one type of five-panel corner element, whereas two types are present in the HMS. As shown in Figure 15a,b, each five-panel corner element, whether in the HUCS or HMS, is composed of a T-shaped unit and a right-angle unit. Consequently, the membrane energy of the five-panel corner element is expressed as [40]

(12) $$E_{m3}=\frac{20M_{o}H^{2}}{t}$$

#### 6.2.4. Six-Panel Corner Element

As illustrated in Figure 15b, the six-panel corner element in the HMS is formed by the rotational superposition of two T-shaped units. Consequently, the membrane energy of the six-panel corner element can be derived by summing the membrane energies of the two constituent T-shaped units. The membrane energy of a single T-shaped unit per folding wavelength is expressed as [41]

(13) $$E_{m4}=\frac{12M_{o}H^{2}}{t}$$

Thus, the membrane energy of the six-panel corner element is

(14) $$E_{m5}=E_{m4}+E_{m4}=\frac{24M_{o}H^{2}}{t}$$

For the HUCS, which contains 4 three-panel right-angle elements (*θ*_1_ = 45°), 28 K-shaped four-panel corner elements (*θ*_2_ = 45°), and 4 five-panel corner elements, the total membrane energy is expressed as

(15) $$E_{m(uc)}=N_{1}E_{m1}+N_{2}E_{m2}+N_{3}E_{m3}=\frac{R_{T1}M_{o}H^{2}}{t}$$

For the HMS, which comprises 8 three-panel right-angle elements (*θ*_1_ = 45°), 136 K-shaped four-panel corner elements (*θ*_2_ = 45°), 20 Type-I five-panel corner elements, 4 Type-II five-panel corner elements, and 4 six-panel corner elements, the total membrane energy is expressed as

(16) $$E_{m(mc)}=M_{1}E_{m1}+M_{2}E_{m2}+(M_{3}+M_{4})E_{m3}+M_{5}\times E_{m5}=\frac{R_{T2}M_{o}H^{2}}{t}$$

where $R_{T}$ is a non-dimensional topological index [43,44].

Substituting Equations (9) and (15) into Equation (5), we obtain

(17) $$F_{mean(uc)}\cdot 2H\cdot k=E_{b(uc)}+E_{m(uc)}=2\pi M_{o}L_{c1}+\frac{R_{T1}M_{o}H^{2}}{t}$$

Thus, we obtain

(18) $$F_{mean(uc)}=\frac{\pi M_{o}L_{c1}}{kH}+\frac{R_{T1}M_{o}H}{2kt}$$

During the crushing process, when the thin-walled structure undergoes ideal deformation under the minimum crushing load, the following expression is derived based on the steady-state condition [35]:

(19) $$\frac{\partial F_{mean(uc)}}{\partial H}=0$$

Consequently, the folding half-wavelength of the HUCS is derived as

(20) $$H=\sqrt{\frac{2\pi tL_{c1}}{R_{T1}}}$$

By substituting Equation (20) into Equation (18), the *MCF* of the HUCS is derived as

(21) $$F_{mean(uc)}=\frac{M_{o}}{k}\sqrt{\frac{2R_{T1}L_{c1}}{t}}$$

For the HMS, substituting Equations (13) and (20) into Equation (5) yields

(22) $$F_{mean(mc)}\times 2H\times k=E_{b(mc)}+E_{m(mc)}=2\pi M_{o}L_{c2}+\frac{R_{T2}M_{o}H^{2}}{t}$$

Thus, we obtain

(23) $$F_{mean(mc)}=\frac{\pi M_{o}L_{c2}}{kH}+\frac{R_{T2}M_{o}H}{2kt}$$

Based on the steady-state condition [35], the following expression is derived:

(24) $$\frac{\partial F_{mean(mc)}}{\partial H}=0$$

Consequently, the folding half-wavelength of the HMS is derived as

(25) $$H=\sqrt{\frac{2\pi tL_{c2}}{R_{T2}}}$$

By substituting Equation (25) into Equation (23), the *MCF* of the HMS is derived as

(26) $$F_{mean(mc)}=\frac{M_{o}}{k}\sqrt{\frac{2R_{T2}\pi L_{c2}}{t}}$$

Equations (21) and (26) are theoretical prediction formulas for the *MCF* of unfilled hierarchical unit-cell and multi-cell thin-walled structures, respectively. Since the focus of this study is on a HUCS and HMS, it is necessary to further derive theoretical formulas for predicting the *MCF* of such foam-filled structures based on foam filling theory and existing studies [42,45,46,47].

The *MCF* of foam-filled square tubes comprises three components: the *MCF* of the empty tube, the uniaxial resistance of the foam material, and the interaction between the tube and foam. The key to predicting energy absorption in foam-filled tubes lies in quantifying the coupling enhancement effect between the foam and the empty tube. Santosa [45] experimentally and numerically investigated foam-filled tubes with plateau stresses ranging from 0.3 to 12.5 MPa. Their study revealed that the coupling enhancement effect between the foam and tube is approximately 0.8 times the energy absorbed by foam compression. Based on this finding, the prediction formula for the *MCF* of foam-filled tubes is expressed as

(27) $$F_{mf}=F_{m}+1.8\sigma_{p}b^{2}$$

Hanssen [46] established an empirical formula for predicting the *MCF* of foam-filled square tubes under quasi-static compressive loading through extensive experimental investigations. The derived formulation is expressed as

(28) $$F_{mf}=F_{m}+\sigma_{p}b^{2}+C\sqrt{\sigma_{p}\sigma_{o}}bt$$

Under dynamic loading conditions, Langseth [42] investigated the dynamic enhancement effects of aluminum alloy thin-walled square tubes, introducing a dynamic enhancement coefficient to account for structural dynamic effects. Building on these findings, Hanssen [46] proposed an empirical formula for predicting the *MCF* of foam-filled tubes under dynamic loading, expressed as

(29) $${F_{mf}}^{d}=\lambda F_{m}+\sigma_{p}b^{2}+C\sqrt{\sigma_{p}\sigma_{o}}bt$$

Under dynamic loading conditions, where *C* is a constant value of 5.5 and λ denotes the dynamic enhancement coefficient accounting for strain-rate effects, the dynamic enhancement coefficient λ for aluminum alloy thin-walled square tubes subjected to loading velocities ranging from 8 m/s to 20 m/s is empirically determined to vary between 1.3 and 1.6 [42]. In this context, three representative values of λ—1.3, 1.35, and 1.4—are selected for analysis. Here, $\sigma_{p}$ represents the plateau stress of the foam material, $\sigma_{o}$ denotes the flow stress of the thin-walled square tube material, and *b* and *t* correspond to the cross-sectional edge length and wall thickness of the square tube, respectively.

By substituting Equation (21) into Equation (29), the *MCF* of the HUCS under axial impact loading can be derived. The governing formula is expressed as

(30) $${F_{mean\left(uc\right)}}^{d}=\lambda \frac{M_{o1}}{k}\sqrt{\frac{2R_{T1}\pi L_{c1}}{t_{1}}}+\sigma_{p}{b_{1}}^{2}+C\sqrt{\sigma_{p}\sigma_{o}}b_{1}t_{1}$$

Similarly, by substituting Equation (26) into Equation (29), the *MCF* of the HMS under axial impact loading conditions can be derived. The corresponding calculation formula is expressed as follows:

(31) $${F_{mean\left(mc\right)}}^{d}=\lambda \frac{M_{o2}}{k}\sqrt{\frac{2R_{T2}\pi L_{c2}}{t_{2}}}+\sigma_{p}{b_{2}}^{2}+C\sqrt{\sigma_{p}\sigma_{o}}b_{2}t_{2}$$

**Figure 16:**
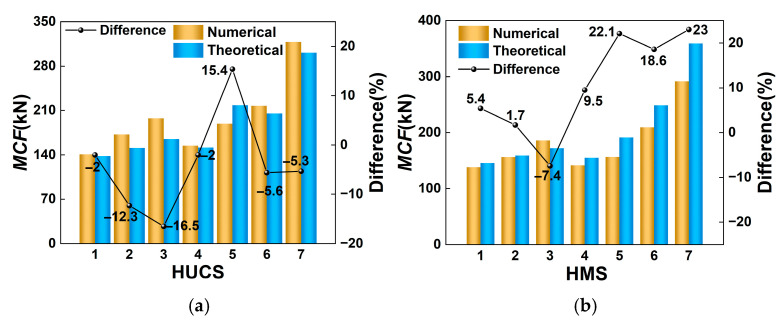
Theoretical analysis of the *MCF* for aluminum foam-filled hierarchical thin-walled structures: (**a**) hierarchical thin-walled unit-cell structure (HUCS), (**b**) hierarchical thin-walled multi-cell structure (HMS).

Based on Equations (30) and (31), the theoretical predictions of *MCF* for the HUCS and HMS under axial impact loading conditions can be calculated. Figure 16 presents comparative analyses between theoretically predicted *MCF* values and numerical simulation results for the HUCS and HMS under axial impact loading. The figure demonstrates that both the HUCS and HMS exhibit minimal discrepancies between theoretical predictions and numerical simulations of *MCF*, thereby confirm the effectiveness of the proposed theoretical model. Furthermore, detailed numerical data presented in Table 5 and Table 6 provide quantitative comparisons of *MCF* values obtained through numerical calculations and theoretical analyses for the HUCS and HMS, respectively. The results indicate that most deviations between numerical and theoretical results remain below 20%, with the minimum observed error reaching 1.7%. This level of agreement is generally reasonable for a simplified analytical model of this complexity, particularly for the baseline configurations. However, it is evident that the predictive accuracy of the proposed model is sensitive to parameter variations. For instance, in the HMS, the model tends to overestimate *MCF* as wall thickness increases (e.g., HMS6 and HMS7). This increasing discrepancy can likely be attributed to the model’s simplified assumptions about the folding mechanism in highly constrained multi-cell sections, where the interaction between adjacent walls and the foam filler may deviate from the idealized SSFE kinematics. Therefore, while the model provides a valuable first-order analytical tool for preliminary design and trend analysis, its application for high-precision predictions in extreme parametric regimes should be approached with caution. It must be re-emphasized that the observed agreement is drawn from a methodologically asymmetric comparison, as detailed in Section 3.2.3. The trends and reasonable deviations observed are encouraging for the preliminary use of the model, but its full predictive capability under high-rate conditions remains to be established with rate-dependent simulations and experiments.

## 7. Conclusions

Based on hierarchical design principles, this study proposed a novel aluminum foam-filled thin-walled structure and conducted theoretical analyses and numerical simulations to investigate the energy absorption performance of a hierarchical unit-cell structure (HUCS) with aluminum foam filling. Building upon this, an aluminum foam-filled hierarchical multi-cell structure (HMS) was developed using a multi-cell collaborative energy absorption strategy. The energy absorption performance of the HUCS and HMS under various impact conditions was explored and compared with that of traditional structures (NHUCS and NHMS). An improved simplified super folding element (*SSFE*) theoretical model, accounting for the constraint-induced strengthening effect of the foam filling, was established to derive a closed-form solution for the mean crushing force (*MCF*) of the HUCS and HMS, and the theoretical predictions were validated against numerical simulation results. The main conclusions are summarized as follows:

1. The novel structures (HUCS and HMS), designed based on hierarchical and multi-cell collaborative principles, exhibit significantly enhanced energy absorption capabilities compared with conventional structures (NHUCS and NHMS). Under equal-mass conditions, the initial peak force (*IPF*) of HUCS-1 and HMS-1 decreased by 75% and 37.5%, respectively, relative to NHUCS-1; compared with NHMS-1, HMS-1 showed an IPF reduction of 20.2%. This comparison verifies that both hierarchical design and multi-cell collaborative design can effectively improve the energy absorption performance of aluminum foam-filled thin-walled structures, with the hierarchical design showing a more pronounced enhancement.
2. During dynamic impact events, both the HUCS and HMS demonstrate a progressive axial-symmetric deformation mode, with force–displacement curves exhibiting the characteristic three-stage behavior (elastic, plateau, and densification). The composite constraint effect generated by the hierarchical and multi-cell designs enhances the stiffness of the structures and triggers the formation of multi-stage plastic hinges, thereby reducing local strain energy density. This mechanism effectively suppresses shear failure of the aluminum foam matrix and mitigates overall structural deformation.
3. By incorporating the constraint-induced strengthening effect of the foam filling, an improved *SSFE* theoretical model was established, from which the closed-form solution for the *MCF* of the HUCS and HMS was derived. The general agreement between theoretical predictions and numerical simulations, with deviations generally below 20% for most parameter ranges, supports the proposed model’s validity as a useful preliminary design tool for estimating *MCF* within the studied parameter range. However, its accuracy diminishes for configurations with large wall thicknesses, where deviations can reach 23%, indicating a need for caution and further model refinement.
4. The energy absorption performance exhibits a strong dependence on wall thickness. Doubling the wall thickness reduces the final deformation by 50.8% for the HUCS and 46.1% for the HMS. In contrast, the influence of aluminum foam density is comparatively weak; doubling the foam density decreases the final deformation by only 17.4% for the HUCS and 3.3% for the HMS. These findings indicate that, although both wall thickness and foam density contribute to improved energy absorption in the HUCS and HMS, wall thickness plays a far more dominant role.
5. For the HUCS and HMS of equal mass, as the impact energy increases, the *IPF* of the HUCS shows an increasing trend while its crushing force efficiency (*CFE*) gradually decreases. In contrast, the HMS exhibits a decreasing *IPF* and a corresponding improvement in *CFE*. This shift is attributed to the distributed yielding mechanism inherent in the multi-cell configuration, suggesting that the HMS may offer superior performance in scenarios involving higher impact energies.

In summary, this study establishes a “hierarchical multi-cell” collaborative design guideline and an *MCF*-based theoretical model, providing an effective strategy for enhancing the energy absorption performance of aluminum foam-filled thin-walled structures. These findings offer a robust theoretical basis and a novel design perspective for the development of high-performance energy-absorbing structures.

## Figures and Tables

**Table 1:** Structural parameters of NHUCS and HUCS.

Specimens	Wall Thickness (mm)	Density of Aluminum Foam (kg/m^3^)	Impact Velocity (m/s)	Height (mm)	Weight (g)
NHUCS1	3.9	234	10	80	214.8
NHUCS2	3.9	234	15	80	214.8
NHUCS3	3.9	234	20	80	214.8
NHUCS4	4.2	337	10	80	248.6
NHUCS5	4.8	526	10	80	310.6
NHUCS6	5.5	234	10	80	283.8
NHUCS7	7.1	234	10	80	352.9
HUCS1	1.3	234	10	80	214.8
HUCS2	1.3	234	15	80	214.8
HUCS3	1.3	234	20	80	214.8
HUCS4	1.4	337	10	80	248.6
HUCS5	1.6	526	10	80	310.6
HUCS6	1.8	234	10	80	283.8
HUCS7	2.3	234	10	80	352.9

**Table 2:** Structural parameters of NHMS and HMS.

Specimens	Wall Thickness (mm)	Density of Aluminum Foam (kg/m^3^)	Impact Velocity (m/s)	Height (mm)	Weight (g)
NHMS1	1.2	234	10	80	214.8
NHMS2	1.2	234	15	80	214.8
NHMS3	1.2	234	20	80	214.8
NHMS4	1.2	337	10	80	248.6
NHMS5	1.2	526	10	80	310.6
NHMS6	1.8	234	10	80	283.8
NHMS7	2.5	234	10	80	352.9
HMS1	0.4	234	10	80	214.8
HMS2	0.4	234	15	80	214.8
HMS3	0.4	234	20	80	214.8
HMS4	0.4	337	10	80	248.6
HMS5	0.4	526	10	80	310.6
HMS6	0.6	234	10	80	283.8
HMS7	0.8	234	10	80	352.9

**Table 3:** Crashworthiness indicators of NHUCS and HUCS.

Specimens	*EA* (J)	*SEA* (J/g)	*F*_max_ (kN)	*F*_mean_ (kN)	*CFE* (%)
NHUCS1	4964.5	23.1	190.4	135.1	70.9
NHUCS2	11,175.4	52.0	193.0	163.4	84.6
NHUCS3	15,674	72.9	193.4	210.8	109.0
NHUCS4	4960.0	19.9	182.8	156.0	85.4
NHUCS5	5053.9	16.3	175.2	186.1	106.2
NHUCS6	4928.0	17.4	206.1	215.0	104.3
NHUCS7	4991.6	14.1	311.1	327.5	105.3
HUCS1	4519.7	21.0	47.7	140.9	295.3
HUCS2	11,374.3	52.9	63.3	172.1	272.1
HUCS3	13,861.6	64.5	109.5	197.6	180.4
HUCS4	4647.7	18.7	43.2	154.5	357.7
HUCS5	5018.2	16.2	55.1	189.2	343.4
HUCS6	5038.3	17.8	72.6	217.4	299.3
HUCS7	5033.6	14.3	92.6	318.3	343.6

**Table 4:** Crashworthiness indicators of NHMS and HMS.

Specimens	*EA* (J)	*SEA* (J/g)	*F*_max_ (kN)	*F*_mean_ (kN)	*CFE* (%)
NHMS1	5013.7	23.3	143.1	119.6	83.6
NHMS2	11,280	52.5	146.9	171.5	116.7
NHMS3	14,049.8	65.4	146.8	199.7	136
NHMS4	5071.9	20.4	146.9	125.5	85.4
NHMS5	5095.5	16.4	126.7	145.7	114.9
NHMS6	5030.7	17.7	218.9	190.7	87.1
NHMS7	5027.3	14.2	241.7	275.7	114.0
HMS1	4567.9	21.3	119.2	138.3	116.1
HMS2	9294.2	43.3	117.2	156.4	131.2
HMS3	12,577.2	58.6	120.5	186.1	154.4
HMS4	4616.3	18.6	122.3	141.7	115.8
HMS5	4998.9	16.1	141.6	156.7	110.7
HMS6	4934.4	17.4	188.8	209.5	110.9
HMS7	5190.4	14.7	234.9	291.9	124.2

**Table 5:** Comparison of numerical and theoretical results of *MCF* for HUCS.

Specimens	*t*_1_ (mm)	Impact Velocity (m/s)	λ	$\sigma_{p}$ (MPa)	Simulation (kN)	Theory (kN)	Error (%)
HUCS1	1.3	10	1.3	2.7	140.9	138.1	−2.0
HUCS2	1.3	15	1.45	2.7	172.1	151.0	−12.3
HUCS3	1.3	20	1.6	2.7	197.6	165.0	−16.5
HUCS4	1.4	10	1.3	3.8	154.5	151.5	−2.0
HUCS5	1.6	10	1.3	8.2	189.2	218.4	15.4
HUCS6	1.8	10	1.3	2.7	217.4	205.3	−5.6
HUCS7	2.3	10	1.3	2.7	318.3	301.3	−5.3

**Table 6:** Comparison of numerical and theoretical results of *MCF* for HMS.

Specimens	*t*_2_ (mm)	Impact Velocity (m/s)	λ	$\sigma_{p}$ (MPa)	Simulation (kN)	Theory (kN)	Error (%)
HMS1	0.4	10	1.3	2.7	138.3	145.7	5.4
HMS2	0.4	15	1.45	2.7	156.4	159.0	1.7
HMS3	0.4	20	1.6	2.7	186.1	172.4	−7.4
HMS4	0.4	10	1.3	3.8	141.7	155.2	9.5
HMS5	0.4	10	1.3	8.2	156.7	191.4	22.1
HMS6	0.6	10	1.3	2.7	209.5	248.5	18.6
HMS7	0.8	10	1.3	2.7	291.9	359.2	23.0

## Data Availability

The original contributions presented in this study are included in the article. Further inquiries can be directed to the corresponding author.

## Nomenclature

NHUCS	Non-hierarchical unit-cell structure
HUCS	Hierarchical unit-cell structure
NHMS	Non-hierarchical multi-cell structure
HMS	Hierarchical multi-cell structure
FEA	Finite element analysis
FE	Finite element
*EA*	Total energy absorption
*SEA*	Specific energy absorption
*F*_max_	Initial peak force (*IPF*)
*SSFE*	Simplified super folding element
2*H*	Folding wavelength
*k*	Effective crushing distance coefficient
*E*_b_	Bending energy
*E*_m_	Membrane energy
$M_{o}$	Full plastic bending moment
$E_{m1}$	Membrane energy of three-panel
$E_{m2}$	Membrane energy of four-panel
$E_{m3}$	Membrane energy of five-panel
$E_{m4}$	Membrane energy of T-shaped-panel
$E_{m5}$	Membrane energy of six-panel
$E_{m(uc)}$	Total membrane energy of HUCS
*CFE*	Crushing force efficiency
η	Impact displacement
*M*	Total mass of structure
*v*	Impact velocity
*E*_total_	Total energy
*E*_KE_	Kinetic energy
*E*_I_	Internal energy
*E*_A_	Hourglass energy
*F*_mean_	Mean crushing force (*MCF*)
$\theta_{i}$	Rotation angle of i-th plastic hinge
$L_{c}$	Total length of all flanges
$\sigma_{o}$	Flow stress of material
$\sigma_{y}$	Yield stress of material
$\sigma_{u}$	Ultimate stress
*θ*_1_	Acute angle of three-panel
*θ*_2_	Acute angle of four-panel
$E_{m(mc)}$	Total membrane energy of HMS
$F_{mean(uc)}$	*MCF* of HUCS under quasi-static compression
$F_{mean(mc)}$	*MCF* of HMS under quasi-static compression
${F_{mean\left(uc\right)}}^{d}$	*MCF* of HUCS under axial impact
${F_{mean\left(mc\right)}}^{d}$	*MCF* of HMS under axial impact
